# Major pathologic response and RAD51 predict survival in lung cancer patients receiving neoadjuvant chemotherapy

**DOI:** 10.1002/cam4.1505

**Published:** 2018-04-19

**Authors:** Apar Pataer, Ruping Shao, Arlene M. Correa, Carmen Behrens, Jack A. Roth, Ara A. Vaporciyan, Ignacio I. Wistuba, Stephen G. Swisher

**Affiliations:** ^1^ Department of Thoracic and Cardiovascular Surgery The University of Texas MD Anderson Cancer Center Houston Texas; ^2^ Department of Thoracic/Head and Neck Medical Oncology The University of Texas MD Anderson Cancer Center Houston Texas; ^3^ Department of Translational Molecular Pathology The University of Texas MD Anderson Cancer Center Houston Texas

**Keywords:** Biomarker, lung cancer, neoadjuvant chemotherapy, RAD51

## Abstract

In a previous study, we determined that major pathologic response (MPR) as indicated by the percentage of residual viable tumor cells predicted overall survival (OS) in patients with non‐small‐cell lung cancer (NSCLC) who received neoadjuvant chemotherapy. In this study, we assessed whether two genes and five protein biomarkers could predict MPR and OS in 98 patients with NSCLC receiving neoadjuvant chemotherapy. We collected formalin‐fixed, paraffin‐embedded specimens of resected NSCLC tumors from 98 patients treated with neoadjuvant chemotherapy. We identified mutations in *KRAS* and *EGFR* genes using pyrosequencing and examined the expression of protein markers VEGFR2, EZH2, ERCC1, RAD51, and PKR using immunohistochemistry. We assessed whether gene mutation status or protein expression was associated with MPR or OS. We observed that *KRAS* mutation tended to be associated with OS (*P *=* *.06), but *EGFR* mutation was not associated with OS. We found that patients with high RAD51 expression levels had a poorer prognosis than did those with low RAD51 expression. We also observed that RAD51 expression was associated with MPR. MPR and RAD51 expression were associated with OS in univariate and multivariate analyses (*P *=* *.04 and *P *=* *.02, respectively). Combination of MPR with RAD51 is a significant predictor of prognosis in patients with NSCLC who received neoadjuvant chemotherapy. We demonstrated that MPR or RAD51 expression was associated with OS in patients with NSCLC receiving neoadjuvant chemotherapy. Prediction of a patient's prognosis could be improved by combined assessment of MPR and RAD51 expression.

## Introduction

Chemotherapy is the foundation of lung cancer treatment. However, many patients who will not benefit from chemotherapy—whether cytotoxic agents or newer targeted therapies—are still exposed to the toxic effects of these drugs. In addition, chemotherapy resistance may develop in patients who receive neoadjuvant chemotherapy, and resistance may contribute to treatment failure in such patients. Establishing prognostic or predictive biomarkers in tissue samples from NSCLC patients treated with neoadjuvant chemotherapy would lead to more accurate prognoses and better identification of patients who may benefit from antitumor therapy. To date, several molecular markers have been proposed as candidate predictors of therapeutic response in patients with NSCLC undergoing neoadjuvant chemotherapy. For example, high ERCC1 expression in resected NSCLC tumors correlated with cisplatin resistance 1.

We previously reported that major pathologic response (MPR) criteria applied to resected tumor specimens of patients who received neoadjuvant chemotherapy can predict survival and be used for assessment of tumor response 2, 3, 4, 5, 6, 7. Our previous study demonstrated that MPR as assessed by the percentage of viable tumor cells in the resected specimen correlated with overall survival (OS) in NSCLC patients who were treated with neoadjuvant chemotherapy 4, 5. We and others also suggested that MPR can be used as a surrogate endpoint for survival, thereby shortening the period needed to evaluate novel chemotherapeutic and biologic therapies in clinical trials 2, 3, 4, 6, 8. The ability to predict tumor response to neoadjuvant chemotherapy using biomarkers will be very helpful for the effective management of NSCLC and for avoiding the development of chemoresistance.

The purpose of this study was to identify biomarkers that predict prognosis or therapeutic response in NSCLC patients treated with neoadjuvant chemotherapy. In this study, we used pyrosequencing to detect mutations in two candidate biomarker genes, KRAS proto‐oncogene (*KRAS*) and epidermal growth factor receptor (*EGFR*). We also used immunohistochemical analysis to examine expression of five candidate protein biomarkers previously identified in the literature: vascular endothelial growth factor receptor 2 (VEGFR2) 9, 10, histone‐lysine‐N‐methyltransferase EZH2 (EZH2) 11, 12, DNA excision repair protein ERCC1 (ERCC1) 1, 13, DNA repair protein RAD51 homolog 1 (RAD51) 14, 15, and PKR^16^, ^17^ in resected tumor specimens from 98 NSCLC patients who were treated with neoadjuvant chemotherapy. Increased VEGFR‐2 gene copy was associated with chemoresistance and shorter survival in patients with non‐small‐cell lung carcinoma who receive adjuvant chemotherapy 9. Researcher suggests that EZH2 may be a predictive and prognostic factor for cisplatin‐based therapy response and disease survival in advanced NSCLC 11, 12. ERCC1 plays a major role in repair of cisplatin‐induced DNA damage in vitro and in vivo 13. Preclinical data suggest that Rad51 might play a role in lung cancer resistance to platinums and etoposide, although this has not been confirmed clinically 18. In NSCLC cell lines, cisplatin exposure increased Rad51 protein induction, and reduction in Rad51 by siRNA significantly increased cisplatin‐mediated cell kill by cisplatin 15, 18, 19, 20. We previously demonstrated that PKR plays a critical role in chemo‐ and radio‐resistance 16, 17, 21. In this study, we found that cytoplasmic RAD51 expression was associated with MPR (higher percentage of viable tumor cells) and shorter OS time in patients with NSCLC receiving neoadjuvant chemotherapy. Combination of MPR with RAD51 is a significant predictor of prognosis in patients with NSCLC who received neoadjuvant chemotherapy.

## Material and Methods

### Patient population

We collected paraffin‐embedded hematoxylin‐ and eosin‐stained slides and blocks from tumors resected from 98 patients with NSCLC. The patients had been treated with neoadjuvant chemotherapy followed by complete surgical resection at The University of Texas MD Anderson Cancer Center from 2008 to 2011. All patients already signed an informed consent form for the use of their clinical data and tumor tissue for molecular research. Detailed clinical and pathologic information, including demographic data, smoking history (never‐ or ever‐smoker), pathologic tumor‐node‐metastasis (TNM) stage, and OS, was available for all patients.

### DNA extraction and mutation analysis

To extract DNA from the formalin‐fixed, paraffin‐embedded (FFPE) tumor specimens, we first placed two to four slices of tumor tissue (10 μm thick) in 1.5‐mL labeled tubes. DNA was purified using a SPRI‐TE Nucleic Acid Extractor (Beckman Coulter, Brea, CA), which uses solid‐phase reversible immobilization technology. For each tumor DNA sample, both the concentration and the quality of the samples were assessed.

To detect gene mutations in the tumor samples, we used pyrosequencing confirmed by direct sequencing. For pyrosequencing, polymerase chain reaction (PCR) amplification was carried out in a 50‐μL reaction tube containing 2 μL of bisulfite‐treated DNA, 5 μL of 10× PCR buffer (Applied Biosystems, Foster City, CA), 2 mmol/L MgCl_2_, 10% dimethyl sulfoxide, 0.2 mmol/L dNTP, 0.25 U of AmpliTaq Gold (Applied Biosystems), 0.1 μmol/L primers for p16, DAPK, RASSF1A, and GSTP1 promoters, 0.01 μmol/L 5′‐tailed, unlabeled forward universal primer or reverse universal primer, and 0.09 μmol/L biotinylated universal primer. PCR products with a 5′‐biotinylated strand were captured on streptavidin‐coated beads (Amersham Biosciences, Uppsala, Sweden). Subsequently, the biotinylated PCR products were purified and made into single‐stranded DNA to which a sequencing primer was annealed using a vacuum prep tool (Pyrosequencing, Inc., Westborough, MA). Pyrosequencing reactions were performed according to the manufacturer's specifications on a PSQHS system (Pyrosequencing AB, Uppsala, Sweden). The *KRAS* exon 1 and exon 2 primers used were as follows: exon 1: forward: 5′‐TCTTAAGCGTCGATGGAGGAG‐3′, reverse: 5′‐TGACATACTCCCAAGGAAAGTAAAG‐3′; exon 2: forward: 5′‐ATGGGTATGTGGTAGCATCTCAT‐3′, reverse: 5′‐AAGTTACTCCACTGCTCTAATCCC‐3′. The *EGFR* primers used were as follows: exon 19: forward: 5′‐TGGTAACATCCACCCAGATC‐3′, reverse: 5′‐ATGAGAAAAGGTGGGCCTGA ‐3′; exon 21: forward: 5′‐CTCAGAGCCTGGCATGAACAT‐3′, reverse: 5′‐CAATACAGCTAGTGGGAAGGC‐3′. For direct sequencing, all PCR amplification products were incubated using exonuclease I and shrimp alkaline phosphatase (Amersham Biosciences, Piscataway, NJ) and sequenced by the MD Anderson Core Sequencing and Microarray Facility.

### Histopathologic evaluation

Immunohistochemical staining for biomarkers was performed as described previously 22. Briefly, FFPE tissue sections (5 μm thick) were deparaffinized, hydrated, and heated in a steamer for 10 min with 10 mmol/L of sodium citrate (pH 6.0) for antigen retrieval. The slides were blocked with 3% H_2_O_2_ in methanol at room temperature for 15 min and then in 10% bovine serum albumin in Tris‐buffered saline with Tween‐20 for 30 min. The slides were then incubated with a primary antibody at 1:400 dilution for 65 min at room temperature. Next, the slides were washed with phosphate‐buffered saline and then incubated with a biotin‐labeled secondary antibody for 30 min. Finally, the samples were incubated with a 1:40 solution of streptavidin‐peroxidase for 30 min. The staining was developed with 0.05% 3′3 diaminobenzidinetetrahydrochloride prepared in 0.05 mol/L of Tris buffer at pH 7.6 containing 0.024% H_2_O_2_. The slides were then counterstained with hematoxylin. An anti‐ERCC1 (8F1) antibody was obtained from Thermo Fisher (Waltham, MA; catalog# MS‐671P). An anti‐EZH2 antibody was obtained from Leica Biosystems (Novocastra Reagents, Buffalo Grove, IL; catalog #NCL‐L‐EZH2). Anti‐FLK‐1 (KDR or VEGFR2, catalog # SC‐6251), anti‐RAD51 (catalog #sc‐8349), and anti‐PKR (SC‐707) antibodies were obtained from Santa Cruz Biotechnology (Dallas, TX).

Immunohistochemical protein expression was quantified using a 4‐value intensity score (0 for negative, 1 for weak, 2 for moderate, and 3 for strong), and the percentage of tumor cells within each category was estimated 23. A final score was obtained by multiplying intensity and extension values (0× % negative tumor cells + 1× % weakly stained tumor cells + 2× % moderately stained tumor cells + 3× % strongly stained tumor cells). The final scores ranged from a minimum of 0 to a maximum of 300.

### Statistical analysis

In the univariate analysis, continuous and categorical variables were analyzed using an independent‐samples *t*‐test or chi‐square test, respectively. The Kaplan–Meier method was used to estimate survival probability as a function of time. Protein expression levels were categorized as either low or high based on a cutoff point set at the median score. A log‐rank test was used to measure between‐group differences in patient survival time. The influence of biomarker expression on survival time was calculated using a multivariate Cox proportional hazards model with adjustment for demographic, clinical, and histopathologic parameters (age, sex, smoking status, and tumor histologic subgroup). A two‐sided *t*‐test was used to test equal proportions between groups in two‐way contingency tables. The generalized estimating equation approach was used to estimate differences in means between groups. Statistical significance was set at *P *<* *0.05.

## Results

### Patient characteristics

Table 1 shows the demographic and clinical characteristics of the 98 NSCLC patients treated with neoadjuvant chemotherapy included in this study. The study population included 54 (55%) men and 44 (45%) women; the patients’ median age was 62 years (range, 41–85 years). The histologic tumor types were adenocarcinoma (*n* = 49), squamous cell carcinoma (*n* = 26), and others (*n* = 23). Most of the patients (*n* = 90, 92%) had received platinum‐based neoadjuvant chemotherapy. The majority of the patients (79 patients, 81%) received a combination platinum‐ and taxane‐based neoadjuvant chemotherapy regimen. The median number of treatment cycles was 3 (range, 2–7 cycles).

**Table 1:** Patient demographics and treatment characteristics

Characteristic	Patients (*N* = 98)
Age (year): mean (range)	62 (41–85)
Gender: *n* (%)	
Male	54 (55%)
Female	44 (45%)
Histology: *n* (%)	
Adenocarcinoma	49 (50%)
Squamous cell carcinoma	26 (27%)
Others^a^	23 (23%)
Tumor size (cm): *n* (%)	
0.0–2.0	14 (14%)
2.1–3.0	24 (26%)
3.1–4.0	30 (30%)
>4.0	30 (30%)
Clinical stage: *n* (%)^b^	
IA/IB	24 (24%)
IIA/IIB	24 (24%)
IIIA/IIIB	46 (47%)
IV	4 (5%)
Pathological stage: *n* (%)	
0/IA/IB	33 (34%)
IIA/IIB	29 (29%)
IIIA/IIIB	33 (34%)
IV	3 (3%)
Neoadjuvant chemotherapy: *n* (%)	
T (Taxol or Taxotere)	80 (82%)
C (Carboplatin or Cisplatin)	90 (92%)
No. of treatment cycles: mean (range)	3 (2–7)

a Others (19 patients with NSCLC‐NOS, four with adenosquamous carcinoma).

b AJCC7.

### Mutation analysis

**Figure 1:**
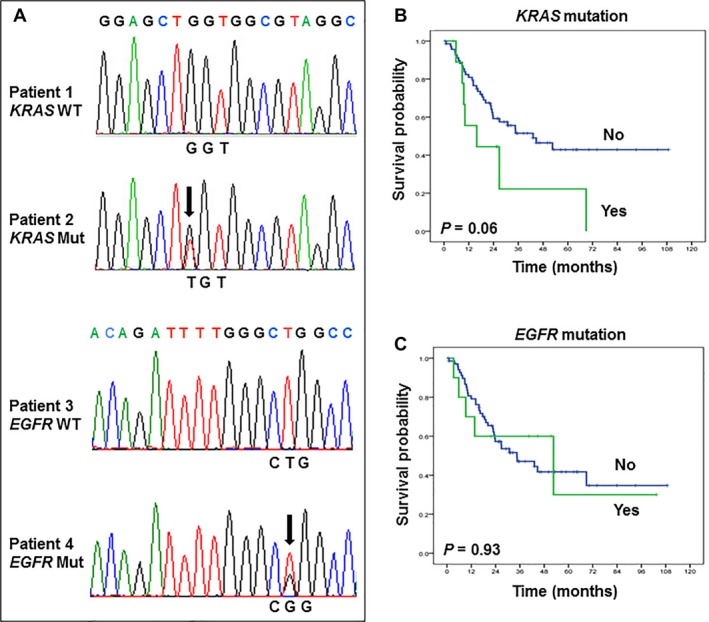
Gene mutation profiles in NSCLC tumors from 98 patients who underwent neoadjuvant chemotherapy. (A) Representative example of wild‐type (WT) and mutated (Mut) *KRAS* and *EGFR*. (B and C) Kaplan–Meier curves comparing overall survival by *KRAS* (B) and *EGFR* (C) mutation status.

We examined *KRAS* and *EGFR* mutations in NSCLC tumors from patients who underwent neoadjuvant chemotherapy. We identified mutations in *KRAS* (codons 12 and 13) and *EGFR* (exons 19 and 21) via pyrosequencing and confirmed these mutations using direct sequencing. The two methods showed similar results. *KRAS* and *EGFR* mutations were detected in samples with a minimum of 32% viable tumor cells (Table 2). In 18 samples with less than 32% viable tumor cells, we detected no mutations, but we found *KRAS* and *EGFR* mutations in 10 of 80 (13%) patient samples with 32% or more viable tumor cells. A point mutation in *KRAS* codon 12 was detected in eight of 80 (10%) samples. All of the *KRAS* mutations detected were in adenocarcinoma specimens. Of the 10 *EGFR* mutations identified, three were a deletion in exon 19, and seven were a point mutation in exon 21. Of the point mutations, three were L858R mutations involving an amino acid substitution from leucine (L) to arginine (R) at position 858 in exon 21. The remaining four point mutations were A859T mutations involving an amino acid substitution from alanine (A) to threonine (T) in exon 21 at position 859. One patient tumor had both *EGFR* and *KRAS* mutations. Figure 1A shows mutation profiles of four patients. Patient 2 had a *KRAS* mutation in codon 12, and patient 4 had an *EGFR* mutation in exon 21. Patients with *KRAS* mutations tended to have shorter OS durations than did patients with wild‐type *KRAS*, but *EGFR* mutation did not affect OS duration (Fig. 1B and C).

**Table 2:** *KRAS* and *EGFR* mutations in NSCLC tumors after neoadjuvant chemotherapy

Patients	Histology	%Viable tumor cells	KRAS mutation	EGFR mutation
1	ADQ	32		Exon 21 (GCC>ACC, A859T)
2	ACC	33	Codon 13 (GGC>GGT)	
3	NSCLC‐NOS	37		Exon21 (CTG>CGG, L858R)
4	ADQ	45		Exon 21 (GCC>ACC, A859T)
5	NSCLC‐NOS	47		Exon 19 (Deletion, E746‐A750)
6	NSCLC‐NOS	47		Exon 21 (CTG>CGG, L858R)
7	ACC	50	Codon 12 (GGT>TGT)	
8	ACC	56	Codon 12 (GGT>TAT)	
9	ADQ	60		Exon 21 (CTG>CGG, L858R)
10	ACC	60	Codon 12 (GGT>GTT)	
11	ACC	61	Codon 13 (GGC>GAT)	
12	ACC	62		Exon 19 (Deletion, L747‐A750)
13	ACC	63	Codon 12 (GGT>GTT)	
14	ACC	68	Codon 12 (GTT>TGT)	
15	ACC	70	Codon 12 (GGT>TGT)	
16	ACC	74		Exon 21 (GCC>ACC, A859T)
17	ACC	75		Exon 19 (Deletion, E746‐A750)
18	ACC	75	Codon 12 (GGT>GTT)	Exon 21 (GCC>ACC, A859T)
19	ACC	81	Codon 12 (GGT>TGT)	

ACC, adenocarcinoma; ADQ, adenosquamous; NSCLC‐NOS, NSCLC‐not otherwise specified.

### Expression of candidate protein biomarkers

**Figure 2:**
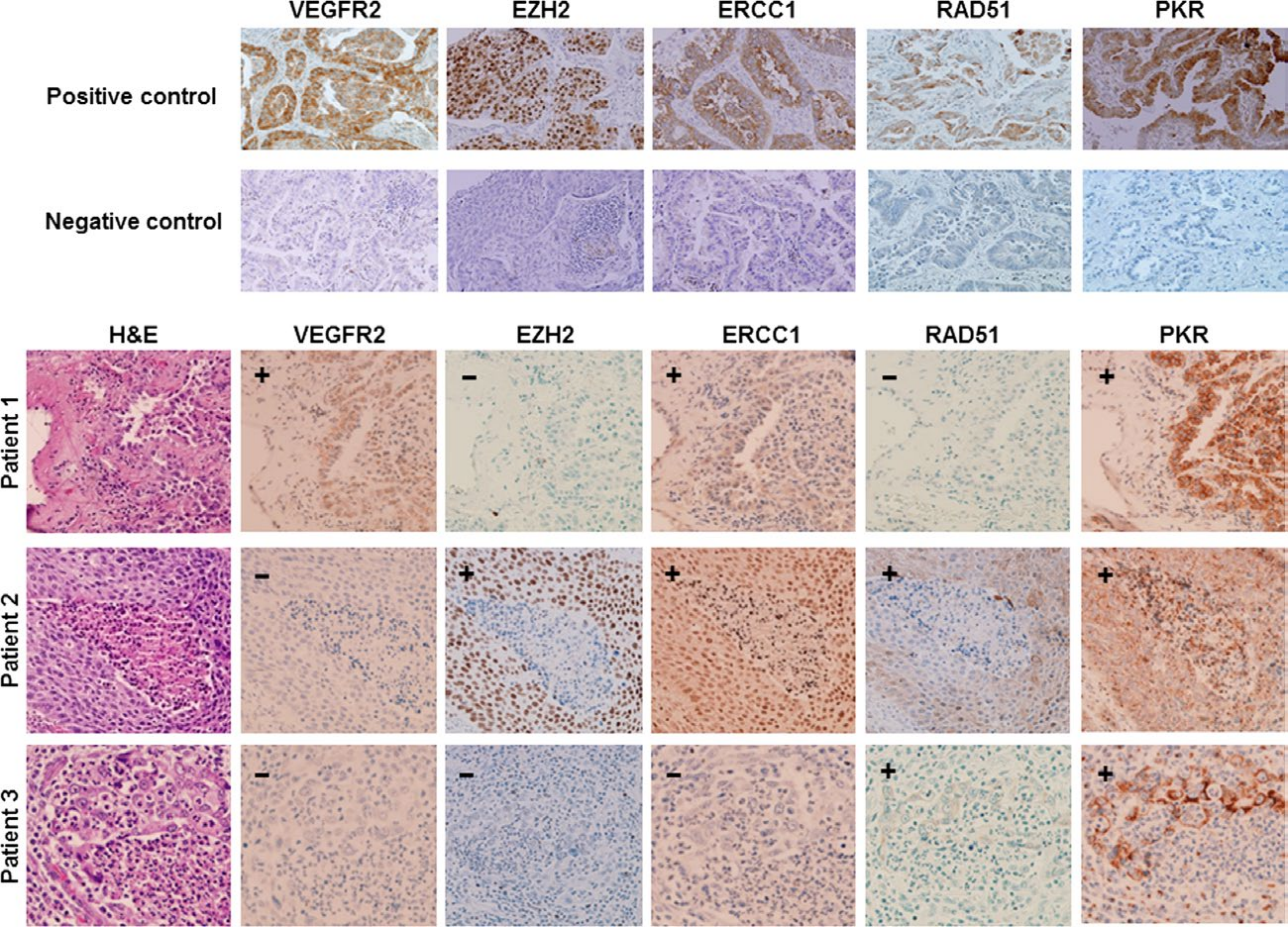
Representative images of VEGFR2, EZH2, ERCC1, RAD51, and PKR expression in NSCLC tumor specimens from patients who received neoadjuvant chemotherapy. (+) indicates positive staining and (−) indicates negative staining.

We next examined the selected protein biomarkers using immunohistochemical analysis. We selected five candidate biomarkers (VEGFR2, EZH2, ERCC1, RAD51, and PKR) on the basis of the literature. Figure 2 shows representative images of VEGFR2, EZH2, ERCC1, RAD51, and PKR staining in NSCLC cells from three patients treated with neoadjuvant chemotherapy. We observed that the VEGFR2 antibody stained the cytoplasm of tumor cells. The EZH2 antibody, in contrast, stained the nucleus of tumor cells. In most samples (96 of 98), the ERCC1 antibody stained the cytoplasm; two samples exhibited predominant ERCC1 cytoplasmic staining with some nuclear staining. Similarly, the RAD51 antibody mainly stained tumor cell cytoplasm; only two samples showed predominant RAD51 cytoplasmic staining with some nuclear staining of tumor cells. The PKR antibody stained tumor cell cytoplasm.

### Correlation of protein biomarker expression with clinicopathologic features and disease outcomes

**Figure 3:**
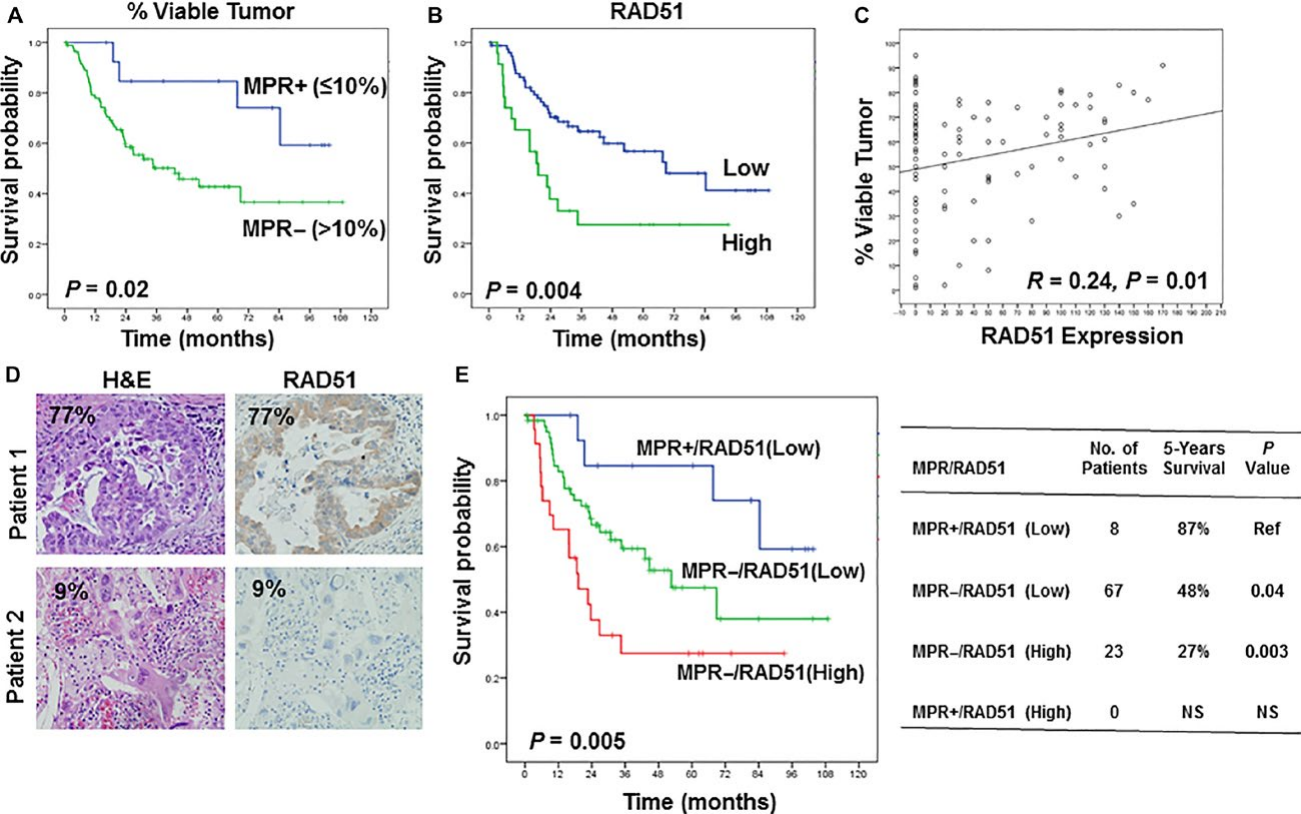
Kaplan–Meier curves showing overall survival by MPR (also indicated as percentage of viable tumor cells) (A) and RAD51 expression level (B). (A) Overall survival was significantly longer in MPR+ patients with ≤10% viable tumor cells than in MPR‐ patients with >10% viable tumor cells. (B) Overall survival was significantly longer in patients with low RAD51 expression than in patients with high RAD51 expression. (C and D) A high percentage of viable tumor cells correlated with high RAD51 expression. H&E, hematoxylin and eosin. (E) Kaplan–Meier curves showing overall survival by combination of RAD51 and MPR. Among patients, the 5‐year overall survival rate in MPR+/RAD51 (High) patients (27%) was significantly lower than that in MPR+/RAD51 (Low) patients (48%) and MPR‐/RAD51 (Low) patients (87%).

Next, we determined whether expression of VEGFR2, EZH2, ERCC1, RAD51, and PKR was associated with MPR and OS time. The surgical pathologic stage, the percentage of viable tumor cells (or MPR), and RAD51 expression were associated with OS in both the univariate and multivariate analyses (Table 3). Figure 3 shows Kaplan–Meier survival curves comparing OS durations by percentage of viable tumor cells (MPR+ vs. MPR−) (Fig. 3A) and by RAD51 expression (Fig. 3B). OS was significantly longer in MPR+ patients who had 10% or less viable tumor cells than in MPR− patients with more than 10% viable tumor cells (*P *=* *.02) (Fig. 3A). We also found that patients with high RAD51 expression levels had a significantly poorer prognosis than did those with low RAD51 expression (*P *=* *.004) (Fig. 3B). RAD51 expression level was also significantly associated with MPR as indicated by the percentage of viable tumor cells (*P *=* *.01) (Fig. 3C). However, we found no association between VEGFR2, EZH2, ERCC1, or PKR expression and MPR (data not shown). We also found no significant relationships between VEGFR2, EZH2, ERCC1, RAD51, or PKR expression and age, sex, tumor status (T status), lymph node status (N status), metastasis status (M status), clinical stage, tumor cell type, or tumor cell differentiation (data not shown). Figure 3D shows representative images of stained tumor tissue from patient 1, with 77% viable tumor cells and high cytoplasmic RAD51 expression, and patient 2, with 9% viable tumor cells and low RAD51 expression in the cytoplasm. We found no associations between the percentage of viable tumor cells or VEGFR2, EZH2, ERCC1, RAD51, or PKR expression and *KRAS* or *EGFR* mutation (data not shown).

**Table 3:** Univariate and multivariate analyses for overall survival in 98 NSCLC patients treated with neoadjuvant chemotherapy

Characteristics	No. of patients	HR (95% CI)	*P*
Univariate analyses			
Age (continuous)	98	1.00 (0.97–1.04)	.830
Gender			
Female (reference)	44	1.00	.070
Male	54	0.5 (0.23–1.07)	
Histology			
Adenocarcinoma (Reference)	49	1.00	.220
Squamous cell carcinoma	26	0.56 (0.26–1.20)	
Other	23	0.62 (0.29–1.31)	
Pathological stage			
0/IA/IB (reference)	33	1.00	.008
IIA/IIB	29	0.73 (0.31–1.72)	
IIA/IIB	33	2.52 (1.27–5.03)	
IV	3	2.78 (0.63–12.35)	
%Viable tumor cells (continuous)	98	1.02 (1.01–1.03)	.004
EZH2 (continuous)	98	1.00 (0.99–1.01)	.510
VEGFR2 (continuous)	98	1.00 (0.99–1.01)	.680
ERCC1 (continuous)	98	0.99 (0.99–1.00)	.650
RAD51 (continuous)	98	1.01 (1.00–1.01)	.02
PKR (continuous)	98	1.00 (0.99–1.01)	.980
%Viable tumor cells			
≤10% (or <=10%) (MPR+) (reference)	8	1.00	.030
>10% (MPR−)	90	3.05 (1.07–8.72)	
RAD51			
Low (reference)	75	1.00	.005
High	23	2.41 (1.31–4.43)	
Multivariate analyses			
Pathological stage			
0/IA/IB (reference)	33	1.00	.007
IIA/IIB	29	0.74 (0.31–1.76)	
IIA/IIB	33	2.63 (0.32–1.76)	
IV	3	2.34 (1.32–5.22)	
%Viable tumor cells (continuous)	98	1.01 (1.00–1.03)	.040
RAD51 (continuous)	98	1.01 (1.00–1.01)	.020
%Viable tumor cells	98		.040
≤10% (or <=10%) (MPR+) (reference)	8	1.00	
>10% (MPR−)	90	2.91 (1.06–7.65)	
RAD51			
Low (reference)	75	1.00	.004
High	23	2.63 (1.35–5.13)	

CI, confidence interval; HR, hazard ratio.

### Prognostic significance of combinations of MPR and RAD51 biomarker

We next further determined whether RAD51 marker provided prognostic information for NSCLC patients treated with neoadjuvant chemotherapy in addition to that provided by MPR. We combined RAD51 and MPR to stratify patients into four groups: MPR+ and RAD51 (Low); MPR+ and RAD51 (High); MPR− and RAD51 (Low); and MPR− and RAD51 (High). Among patients, the 5‐year overall survival rate in MPR+/RAD51 (High) patients (27%) was significantly lower than that in MPR+/RAD51 (Low) patients (48%) and MPR−/RAD51 (Low) patients (87%) (Fig. 3E). We did not observe any patients in second group: MPR+ and RAD51 (High) (Fig. 3E). Our results also revealed that the MPR/RAD51 was significantly associated with prognosis and was an independent indicator of survival duration in NSCLC patients treated with neoadjuvant chemotherapy.

## Discussion

The significance of mutations in *KRAS*,* EGFR*,* ALK*,* ERBB2/HER2*,* PI3KCA*, and *BRAF* has been documented in primary NSCLC tumors 24. However, only a limited number of studies have investigated gene mutations in NSCLC tumors that have been previously treated with neoadjuvant chemotherapy 25. In this study, we first investigated *EGFR* and KRAS gene mutations in NSCLC tumors treated with neoadjuvant chemotherapy. We observed no association between *EGFR* mutation and OS or MPR. However, we observed that *KRAS* mutation was associated with OS. We further evaluated the ability of five candidate markers (VEGFR2, EZH2, ERCC1, RAD51, and PKR) to predict prognosis and therapeutic response. We demonstrated that cytoplasmic RAD51 expression was associated with both MPR (as indicated by the percentage of viable tumor cells) and OS. We found that patients with high RAD51 expression levels had a poorer prognosis than did those with low RAD51 expression. Our results suggest that RAD51 expression in the cytosol is a useful prognostic biomarker in patients with NSCLC who have undergone neoadjuvant chemotherapy.

Our results indicated that the MPR in the resected specimen may serve as a surrogate endpoint for survival to evaluate novel chemotherapeutic therapies and immunotherapy response in biomarker‐driven translational clinical trials. Assessment of biomarker could be combined with MPR to accurately serve as surrogate endpoints for treatment efficacy. One potential limitation of our study is that we did not compare pretherapy and post‐therapy tissue specimens from patients whose tumors did not respond to neoadjuvant therapy. Unfortunately, we were unable to collect FFPE biopsy specimens from these patients, so we could not compare them with post‐therapy tissues from the same patients.

Increased RAD51 expression has been shown to be associated with poorer outcomes in patients with several tumor types treated with chemoradiotherapy 14, 15, 18, 19, 20, 26. Furthermore, a number of reports demonstrated that RAD51 is involved in resistance to anticancer treatments such as radiation and platinum chemotherapy agents in various tumor types, including lung cancer 14, 15, 19, 20, 26. For instance, silencing the *RAD51* gene improved sensitivity to doxorubicin in soft tissue sarcoma cell lines 14. Downregulation of RAD51 expression by gefitinib (a selective EGFR tyrosine kinase inhibitor) sensitized mitomycin C and gemcitabine‐induced cell inhibition in lung cancer cells 26, 27.

RAD51 plays a critical role in a common DNA damage response pathway associated with the activation of homologous recombination and double‐strand break repair 14, 15, 26. In the nucleus, RAD51 binds to single‐ and double‐stranded DNA and exhibits DNA‐dependent ATPase activity 15. In the cytoplasm, RAD51 is involved in maintenance of the mitochondrial genome 18. Cytoplasmic RAD51 plays important roles in maintaining the integrity of mitochondrial DNA and facilitating its repair 18. Several studies have indicated that RAD51 protein can translocate between cytoplasmic and nuclear compartments 14, 15, 26. Several other proteins have recently been found to be involved in mitochondrial DNA repair, including aprataxin 28, tyrosyl‐DNA phosphodiesterase 1 (TDP1) 28, and flap endonuclease 1 (FEN1) 29. Aprataxin is involved in the repair of DNA strand breaks caused by various DNA‐damaging agents, including H_2_O_2_, methyl methane sulfonate, and the irinotecan‐related compound camptothecin 30. High levels of aprataxin expression are associated with poor response to irinotecan‐based chemotherapy 30. TDP1 has been linked with resistance to camptothecin and a topoisomerase I inhibitor in human lung cancer 31. Several studies have demonstrated that downregulation of overexpressed FEN1 using a short interfering RNA or an inhibitor increased sensitivity to cisplatin in brain, lung, and gastric cancer cells 32, 33, 34. Further study is needed to explore other candidate markers in existing FFPE tissue from NSCLC patients treated with neoadjuvant chemotherapy. Understanding the mechanisms of interaction of biomarkers will clarify their contribution to chemoresistance and may lead to the recognition and use of these markers in clinical practice.

In conclusion, we demonstrated that high cytoplasmic RAD51 expression was associated with MPR (as indicated by the percentage of viable tumor cells) and shorter OS in patients with NSCLC receiving neoadjuvant chemotherapy. Combination of MPR with RAD51 is a significant predictor of prognosis in patients with NSCLC who received neoadjuvant chemotherapy. Prediction of a patient's prognosis could be improved by combined assessment of standard clinical variables, MPR, and molecular biomarkers.

## Conflict of Interest

The authors declare no conflict of interests.
